# The effects of cichorium intybus extract on the maturation and activity of dendritic cells

**DOI:** 10.1186/2008-2231-22-28

**Published:** 2014-02-24

**Authors:** Mohammad Hossein Karimi, Salimeh Ebrahimnezhad, Mandana Namayandeh, Zahra Amirghofran

**Affiliations:** 1 Transplant Research Center, Shiraz University of Medical Sciences, Shiraz, Iran; 2 Department of Immunology, Autoimmune Disease Research Center and Medicinal and Natural Products Chemistry Research Center, Shiraz University of Medical Sciences, Shiraz, Iran

**Keywords:** DCs, Cichorium intybus, Dendritic cells, Immunomodulation, T cell responses

## Abstract

**Background:**

*Cichorium intybus* is a medicinal plant commonly used in traditional medicine for its benefits in immune-madiated disorders. There are several evidences showing that *C. intybus* can modulate immune responses. In the present study we have investigated the effects of the ethanolic root extract of this plant on the immune system by targeting dendritic cells (DCs). For this purpose, phenotypic and functional maturity of murine DCs after treatment with the extract was analyzed by flow cytometry and mixed lymphocyte reaction (MLR) assay.

**Results:**

*C. intybus* did not change the expression of CD40, CD86 and MHC-II molecules as important co-stimulatory markers on DCs compared to the control, indicating that it could not promote DCs phenotypic maturation. Treatment of DCs with lower concentrations of the extract resulted in an increased production of IL-12 by these cells with no change in IL-10 release. The capacity of treated DCs to stimulate allogenic T cells proliferation and cytokines secretion was examined in the co-cuture of these cells with T cells in MLR. *C. intybus* at higher concentrations inhibited proliferation of allogenic T cells and in lower concentrations changed the level of cytokines such that IL-4 decreased and IFN-γ increased.

**Conclusions:**

These results indicated that *C. intybus* extract at higher concentrations can inhibit T cell stimulating activity of DCs, whereas at lower concentrations can modulate cytokine secretion toward a Th1 pattern. These data may in part explain the traditional use of this plant in treatment of immune-mediated disorders.

## Introduction

The use of herbal medicine is increasing in therapies of immune disorders, including autoimmune diseases and cancers in all over the world. *Cichorium intybus (C. intybus*) belonging to Asteraceae family, also known as chicory, grows as a wild plant and is a well-known herb with various biological activities. This plant is native to Europe and Asia and has been widely used in folk medicine for treatment of gallstones, appetite loss, gout, jaundice, skin swellings, rheumatism and liver inflammation [1,2]. The seed extract of *C. intybus* has shown high antioxidant activity, short- and long-term beneficial effects on diabetes [3] as well as ameliorating effects on non-alcoholic fatty liver disease [4]. Methanolic extract of *C. intybus* and its various fractions have revealed wound healing effects. β-Sitosterol has been considered as a main component of chicory extract in wound healing [5]. The methanolic extract of the plant has demonstrated some anticancer and apoptosis inducing effects [5]. *C. intybus* could ameliorate the oxidative stress, hepatic injury and cellular damage induced by chemical compounds in rat [6,7]. In addition, this plant has shown anti-inflammatory activity by inhibiting TNF-α mediated inflammation and reducing cyclooxygenase (COX)-2 protein expression [1]. The ethanolic root extract of *C. intybus* has inhibited mitogen-activated human lymphocyte proliferation as well as allogenic T cell responses [8] which implies the ability of this plant to modulate immune responses.

Dendritic cells (DCs), as the most potent antigen presenting cells for naïve T cells, act as a link between the acquired and innate immune systems and are responsible for the initiation of the protective immune response as well as the induction of immune tolerance [9]. The function of these cells is affected by their maturation status, origin and phenotype [10]. These cells have the unique ability to stimulate and target naive T cells to either Th1 or Th2 cells [11]. They can also effectively down-regulate T-cell responses through the generation of T regulatory cells [12]. DCs in immature forms can effectively present antigen but because of low expression of co-stimulatory molecules such as CD86, CD40 and MHC II they cannot properly stimulate immune system [10]. T cells activation and proliferation are inhibited and suppressed with repeated stimulation by immature DCs. Maturation of DCs converts them to the cells that can stimulate immune system vigorously. Therefore inhibition of this process is a valuable strategy to modulate immune responses. In this regard, using DCs with low expression of co-stimulatory molecules can be considered as a beneficial approach in therapy of autoimmune disease and transplantation [13]. For this reason, considerable studies have been performed to use these cells as therapeutic targets for immunomodulatory effects of some pharmacological compounds.

Given the important role of DCs in immune response as well as immune regulation and the presence of data regarding the immunomodulatory effects of *C. intybus*, we hypothesized that this plant might have some modulatory effects on DCs. To the best of our knowledge, there is no study about the effect of *C. intybus* on DCs. Therefore in the present study we have investigated the effects of the root extract of this plant on the maturation and function of DCs through evaluation of the expression of DC maturation markers, their allostimulatory capacity and release of the main Th1 and Th2 cytokines.

## Materials and methods

### Animals

6- week-old male BALB/c and C57BL/6 mice were purchased from Razi Institute (Shiraz, Iran) and were kept under optimal conditions of hygiene and received standard mouse chow and water *ad libitum*. All experimental procedures on handling the animals were approved by the ethical committee of Shiraz University of Medical Sciences.

### Purification of splenic DCs

In order to isolate DCs from spleen, the gradient media (Nycodenz, Axis Shields, Norway) was used as previously described [14]. Briefly, mice spleens were chopped and digested with 1 mg/ml collagenase D (Roche, Germany) and 0.02 mg/ml DNase (Roche) and meshed with 0.2 μm sieve. Cells were washed with RPMI 1640 culture medium (Sigma, St. Louis, MO, USA) containing 5 mM EDTA. The pellet was resuspended in culture medium with 10% fetal calf serum (FCS) and 5 mM EDTA. The cell suspension was layered on Nycodenz 12.5% (w/v), d = 1.068 and centrifuged at 1800 rpm and 4°C for 20 min. The interface layer was collected and washed two times and 1 × 10^4^ cells cultured in 3 cm plate for 2 h at 37°C in a 5% CO_2_ incubator. After that, non-adherent cells were discarded by washing and adherent cells were used for tests. Purity of the adherent cells was determined with analysis for the expression of CD11c molecule by flow cytometry. The cells were routinely more than 90% CD11c positive.

### Preparation of the ethanolic root extract of *C. intybus*

The roots of *C. intybus* were collected from Fars provinces at October and authenticated by Mr Iraj Mehregan, from Shiraz School of Pharmacy. A voucher specimen was deposited in the Herbarium of the School of Pharmacy. Samples were washed, dried and then 200 g of each shade dried powder was extracted in a percolator containing 70% ethanol. After 72 h, percolation was done and the extract solution was concentrated in a rotary evaporator (Heidolph, Germany). The dried extract was dissolved in dimethyl sulphoxide (DMSO) and then resuspended in RPMI 1640 medium to obtain 20 mg/ml solution.

### Treatment of DCs with the extract

DCs were treated with *C. intybus* extract at final concentrations of 0.1, 1, 10 and 100 μg/ml in the cell culture plates. As negative control, cells were treated with the vehicle (DMSO) at the highest concentration used in the tests (0.1%) and as positive control, cells were treated with TNF-α (Sigma), the known DC maturation inducing cytokine, at concentration of 40 ng/ml.

### MTT cell viability assay

In vitro cytotoxicity of the extract on DCs was tested by 3-(4, 5-dimethylthiazol-2-yl)-2, 5-diphenyltetrazolium bromide (MTT) colorimetric assay as described previously [15]. DCs were treated with different concentrations of the extract ranging from 0.1 to 100 μg/ml for 24 h, then 10 μl MTT (5 mg/ml, Sigma) was added to each well and cells were incubated for an additional 4 h at 37°C. The optical density (OD) of each well was measured at 570 with reference at 630 nm on an enzyme-linked immuosorbent assay (ELISA) plate reader. The viability was determined as the follows: (OD of extract-treated cells/OD of DMSO-treated cells) × 100.

### Flow cytometry analysis

Isolated DCs were treated with non toxic concentrations of the extract for 18 h and were then analyzed for the expression of co-stimulatory molecules in a flow cytometer (FACSCalibur, Beckton Dickinson Biosciences, San Jose, CA). Cells were stained with phycoerithrin (PE)-conjugated anti-CD11c, fluorescence isothiocyanate (FITC)-conjugated anti-CD40, FITC-conjugated anti-CD86 and FITC-conjugated anti-MHC II antibody and appropriate conjugated isotypes all from Beckton Dickinson (BD) Pharminogen (San Diego, CA). Data were analyzed using Win MDI software (Scripps, La Jolla, CA). The ratio between the percentage of markers expression on extract-treated DCs and DMSO-treated DCs was calculated. The mean florescent intensity (MFI) of the expression of markers on extract-treated DCs were also determined and compared with DMSO-treated DCs.

### Allogeneic mixed lymphocyte reaction (MLR)

In order to evaluate the proliferative effect of extract-treated DCs on T lymphocytes, MLR assay was used. For this, T cells were purified from lymph nodes of C57BL/6 mice using nylon wool. The purity was determined using FITC-conjugated anti-CD3 antibody (BD Pharminogen) by flow cytometry. *C. intybus*-treated DCs were inactivated with mitomycin C (0.5 mg/ml) for 20 min, then cells were washed with phosphate buffered saline (PBS) for three times and resuspended in culture medium containing 10% FCS. For MLR assay, 10^4^cells/well mitomycin-treated DCs, as stimulator cells, were added in a 96-well round-bottomed culture plate (Nunc, Denmark) in triplicates and co-cultured with 10^5^ allogenic T cells, as responder cells, for 48 h. A triplicate wells containing DMSO-treated DCs plus allogenic T cells were used as negative control. T cell proliferation was measured by a 5-Bromo-20-deoxy-uridine (BrdU) cell proliferation assay kit (Roche, Germany) according to the manufacturer’s instructions. Proliferation was determined as the follows: (OD of extract-treated culture/OD of DMSO-treated culture) × 100.

### Cytokines assay

The supernatant of extract-treated DCs and MLR cultures were collected and used to measure IL-12, IFN-γ, IL-10 and IL-4 by ELISA kits according to the manufacturer’s protocol (eBioscience, USA). The sensitivity of IL-4, IFN- γ, IL-10 and IL-12 kits were 4, 15, 30 and 15 pg/ml, respectively.

### Statistics analysis

All data were representative of at least three or two independent experiments performed in triplicate and presented as mean ± standard deviation (SD). The differences between groups were analyzed by Student’s *t*-test and oneway ANOVA using Graph-Pad Prism 5 software (Graph-Pad Software Inc, San Diego, CA). P vales less than 0.05 were considered significant.

## Results

### Effects of *C. intybus* on viability of DCs

**Figure 1:**
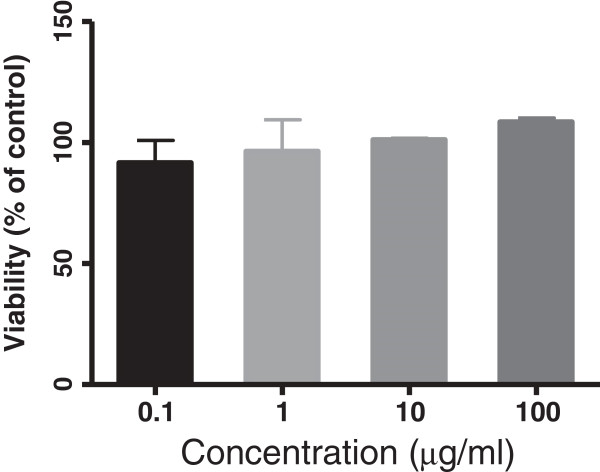
**Cell viability assay of *C. intybus *ethanolic extract on DCs after 24 h treatment determined by MTT assay.** Control was DCs treated with DMSO at the highest concentrations used in the tests (e.g., 0.1). The bars indicate mean ± standard deviation of three independent experiments performed in triplicate. The extract had no significant growth inhibitory effects on DCs.

In order to determine the effects of *C. intybus* on the viability of DCs, these cells were treated with different concentrations of the plant extract for 24 h and then MTT assay was performed. The results showed that this extract at concentration of 0.1, 1, 10 and 100 μg/ml had no cytotoxic effect on DCs (Figure 1), therefore these concentrations were used for the next experiments on DCs.

### Effect of *C. intybus* on maturation of mouse splenic DCs

**Figure 2:**
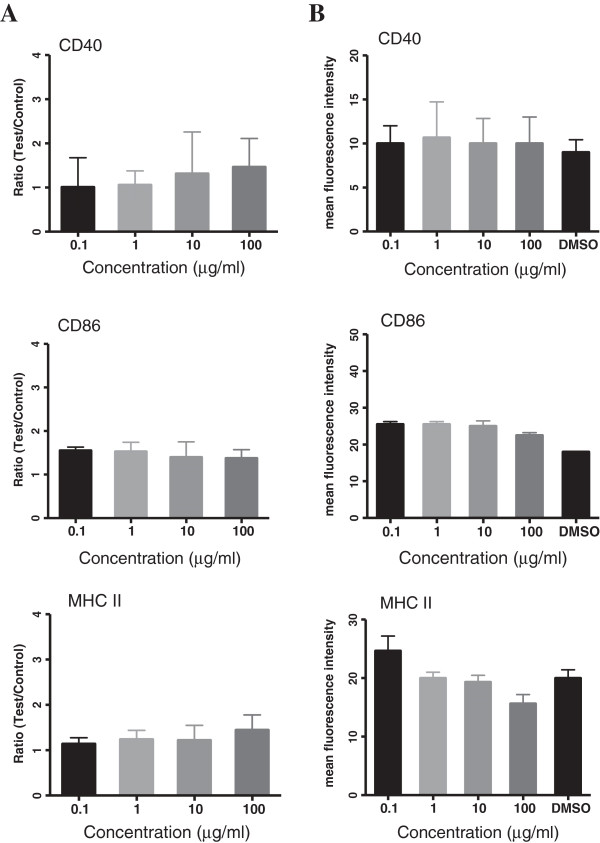
**Effect of *C. intybus *ethanolic extract on phenotypic maturation of DCs.** DCs were treated with the extract for 18 h and then the expression of CD40, CD86 and MHC II molecules was determined by flow cytomety. Negative control was DCs treated with DMSO. **A)** The bars indicate mean ± SD of the ratio between the percentage of markers expression on DCs treated with the extract and those treated with DMSO. **B)** The bars indicate mean ± SD of the mean fluorescence intensity (MFI) of the expression of markers. No significant difference in the ratio and MFI of the markers expression between extract-treated DCs and control cells was observed.

*C. intybus*-treated DCs were analyzed by flow cytometry for the expression of CD40, CD86 and MHC II co-stimulatory molecules. As data in Figure 2A shows, the extract did not significantly modulate the percentage expression of these molecules at concentration of 0.1 to 100 μg/ml (see Additional file 1: Figure S1 for dot plots). Although, an increasing concentrations of the extract has led to a decreasing MHC II fluorescence intensity of expression (p < 0.01), none of the molecules MFI was significantly different between the extract-treated cells and the corresponding control (Figure 2B).

### Effect of *C. intybus* treated DCs on proliferation of T cells

**Figure 3:**
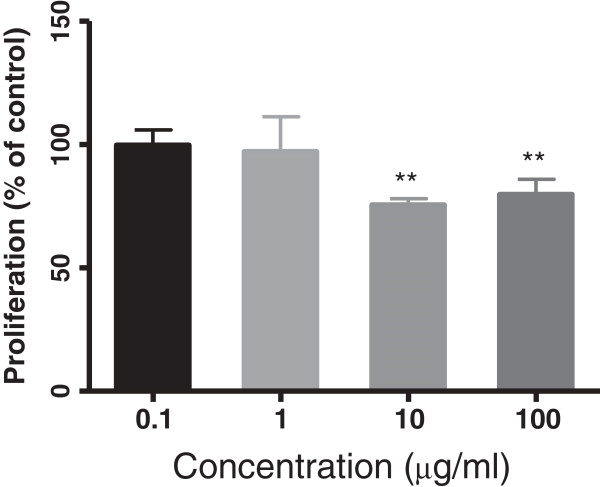
**The effect of DCs treated with *C. intybus *extract on T cells proliferation in MLR assay.** DCs were treated with the extract for 18 h and then co-cultured with allogenic T cells for 48 h. Control was DMSO-treated DCs plus T cells. Cell proliferation was measured by Brdu incorporation assay. The bars indicate mean ± SD of the cell proliferation in the presence of the extract as compared to the proliferation of controls taken to be 100%. DCs treated with 10 and 100 μg/ml of extract have decreased T cells proliferation (**P < 0.01).

In order to find the effects of the plant on DCs function, mouse splenic DCs were treated with concentrations of 0.1 to 100 μg/ml of the extract for 18 h and then cells were co-cultured with allogenic T cells in MLR assay. The proliferation of T lymphocytes was evaluated using BrdU incorporation assay. As results in Figure 3 show, ethanolic extract of *C. intybus* decreased the proliferation of T cells at higher concentrations. The proliferation of these cells decreased to 75.60 ± 2.5 and 79.80 ± 6.1 percent of control when DCs had been treated with 10 and 100 μg/ml of the extract, respectively (P < 0.05).

### Effect of *C. intybus*-treated DCs on the production of cytokines

**Figure 4:**
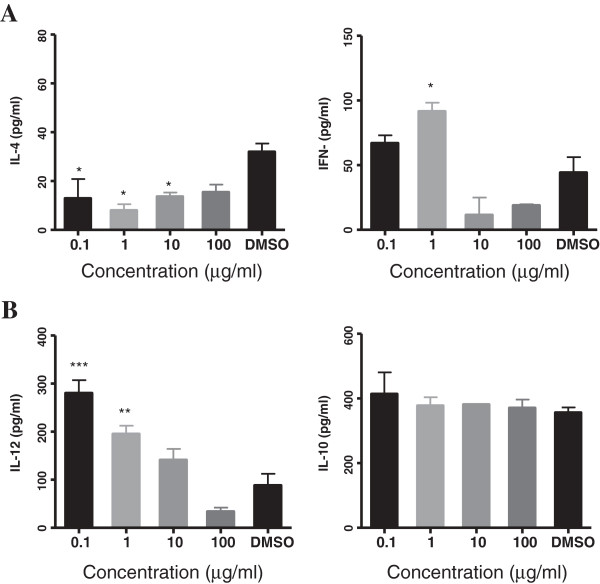
**Effect of *C. intybus *extract on cytokine production. (A)** The effect of the extract on IL-4 and IFN- γ production in MLR assay. Control was DMSO-treated DCs plus allogenic T cells. **(B)** The effect of the extract on IL-12 and IL-10 secretion by extract treated-DCs after 18 h. Control was DMSO- treated DCs. *P < 0.05, **P < 0.01, ***P < 0.001 shows significant difference with the negative control.

The effect of the extract on IL-4 and IFN- γ production in MLR is demonstrated in Figure 4A. A decreased IL-4 level in the supernatant of T cells co-cultured with DCs treated with lower concentrations of *C. intybus* in comparison with the control was observed (P < 0.05). The level of this cytokine was 12.98 ± 7.87 and 8 ± 2.51 pg/ml at concentrations of 0.1 and 10 μg/ml of the extract, respectively, compared to the control (32.04 ± 3.35 pg/ml). Conversely, *C. intybus* at concentration of 1 μg/ml significantly increased IFN-γ production in the supernatant of MLR culture in comparison with the control.

### Effects of *C. intybus* on IL-12 and IL-10 production by DCs

The level of IL-12 and IL-10 in the supernatant of extract-treated DCs was measured. As the result in Figure 4B shows, IL-12 level was significantly higher at concentrations of 0.1 (280.6 ± 26.58 pg/ml) and 1 μg /ml of the extract (195.5 ± 16.88 pg/ml) than the negative control (88.58 ± 23.87 pg/ml, P < 0.05). The level of IL-10 didn’t show any significant differences in the supernatant of extract-treated and DMSO-treated DCs.

## Discussion

In various studies the ability of plants and their derivatives to modulate DCs and induce changes in the expression of co-stimulatory molecules, cytokine secretion patterns and their T cell stimulating activity have been investigated [16,17]. DCs, the key cells of antigen presentation are considered as the important targets for immune response as well as immune regulation.

*C. intybus* has been widely used as a remedy for treatment of various inflammatory diseases. There are several reports about its pharmacological actions and anti-inflammatory effects [1,7]. The root extracts of this plant have shown anti-inflammatory properties in animal models of arthritis. Moreover, in a clinical trial, the safety and usefulness of a proprietary bioactive extract of its root in patients with osteoarthritis have been demonstrated [18]. A decrease observed in macrophage migration inhibitory factor (MIF) serum level in healthy volunteers consuming Chicory coffee [19] and an inhibition in the expression and activity of COX-2 by the ethyl acetate root extract in human colon carcinoma HT29 cells treated with the pro-inflammatory cytokine TNF-α are further evidences implying the effectiveness of this plant on modulation of immune mediators release [1].

As DCs have a critical role in inducing inflammation as well as immune responses, in the present study we arranged a set of experiments to examine the effect of the plant extract on maturation and function of splenic DCs. The phenotypic maturation of DCs was investigated via evaluation of the expression of CD40, CD86 and MHC- ІІ molecules which are important co-stimulatory markers on DCs and have critical roles in antigen presentation and T cell activation. The extract revealed no significant effect on the expression of CD40 and CD86 molecules, showing that it could not promote DCs maturation. The expression of MHC II antigen on DCs was not also significantly different with the control, however the intensity of expression of this molecule showed a significant decrease by increasing the concentration of the extract up to 100 μg/ml, indicating a dose-dependent trend to reduce the expression of this molecule on treated DCs as the extract concentration is increased. Any decrease in the expression of the co-stimulatory molecules like MHC II on DCs can be resulted in failure of such DCs to provide an effective response for T cells because these DCs are not only in an immature state but may also have acquired a tolerogenic feature. As result of our study showed, the extract at higher concentrations decreased the proliferation of T cells in allogenic response which is in line with the above results and suggests the ability of the extract to affect T cell signaling activity of DCs.

A major characteristic of DCs is synthesis and release of cytokines with modulatory functions during inflammation and T cell differentiation. Immune response is skewed to humoral or cellular immunity based on cytokines secreted by DCs. IL-12 is a cytokine expressed and released by DCs which can induce differentiation of T cells to Th1 and cellular immunity, by contrast IL-10 is a Th1 inhibitory cytokine [20,21]. An elevated IL-12 secretion level by DCs at concentrations of 0.1 and 1 μg/ml of the extract and no change in IL-10 release was observed in this study. As IL-4 and IFN-γ are landmark of deviation to Th1 or Th2, these cytokines were measured in the supernatant of MLR assay. The results indicated a decrease in IL-4 production versus an increase in IFN-γ secretion at concentrations of 0.1 and 1 μg/ml of the extract. In a normal immune response, splenic DCs expressing IL-12 in co-culture with T cells can induce production of IFN-γ and deviation to Th1 while neutralizing of IL-12 inhibit Th1 response and increase Th2 response [22]. Therefore, the increased IL-12 production by DCs when they were treated with low concentrations of the extract along with production of more IFN-γ and less IL-4 by T cells in MLR suggest the ability of *C. intybus* extract to deviate the cytokine pattern of T cells toward a Th1 response. Of note, the extract at concentration of 100 μg/ml has reduced both IL-12 secretion by treated DCs and IFN-γ release in MLR, which indicated the inhibitory effect of the extract at higher concentration on the immune response. It is reasonable to assume that the decreasing trend observed in the expression of MHC II molecules on DCs, as mentioned before might be a reason for the diminished T cells proliferation and cytokines release observed at higher concentration of the extract. To confirm Th1 polarization in co-culture of T cells with *C. intybus* treated-DCs, study of the expression of T bet and GATA-3 as related transcription factors for Th1 and Th2 differentiation is recommended [23]. Moreover, the difference between the effect of the extract at lower and higher concentrations may be attributed to the presence of various constituents in the extract with different mode of actions. As we used ethanolic crude root extract of the plant, it would be necessary to identify the compound/s responsible for the observed effects in future studies.

## Conclusions

*C. intybus* extract at higher concentrations inhibited T cell stimulating activity of DCs. In contrast, at lower concentrations the extract increased IL-12 production by DCs and modulated cytokine release of T cells toward a Th1 pattern.

Based on these findings further in vivo studies to elucidate the effect of these compounds on the complex pathways of DC regulation via IFN-γ and IL-4 as well as their effects on other T cell subsets such as T regulatory cells may lead to the development of clinical applications exploiting these compounds for the treatment of various immune diseases.

## Competing interests

The authors declare that they have no competing interests.

## Authors’ contributions

ZA provided plant materials and with MHK designed and supervised the study and finalized the manuscript. SE carried out the experiments and prepared the draft of the manuscript. All authors read and approved the final manuscript.

## Supplementary Material

Additional file 1: Figure S1 Effect of *C. intybus* ethanolic extract on phenotypic maturation of DCs. Click here for file
