# Supplementary material for: The effects of cichorium intybus extract on the maturation and activity of dendritic cells
Source: Daru. 2014 Feb 24;22(1):28. doi: 10.1186/2008-2231-22-28 (PMC3936942; doi:10.1186/2008-2231-22-28)
Supplement: Additional file 1: Figure S1 — Effect of C. intybus ethanolic extract on phenotypic maturation of DCs. [file 2008-2231-22-28-S1.doc]

**Additional file 1: Figure S1**

**Effect of *C. intybus* ethanolic extract on phenotypic maturation of DCs.** DCs were treated with the extract for 18 h and then the expression of CD40, CD86 and MHC II molecules was determined by flow cytomety. Negative control was DCs treated with DMSO. Dot plots indicate the percentage of markers expression on CD11c positive DCs. The result shown is one representative experiment out of three independent experiments.

|  | CD40 | CD86 | MHC II |
| --- | --- | --- | --- |
| 0.1  µg/ml | 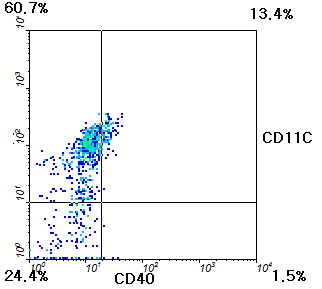 | 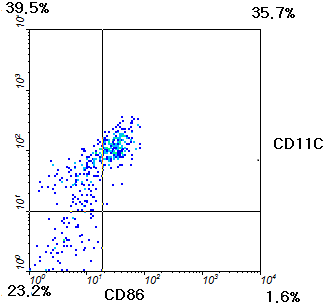 | 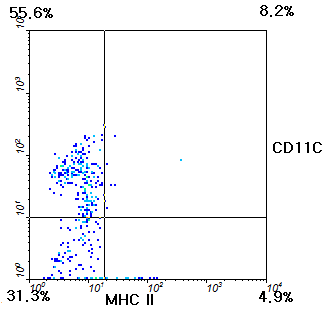 |
| 1  µg/ml | 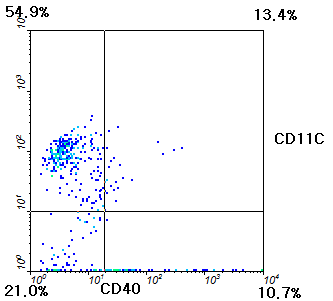 | 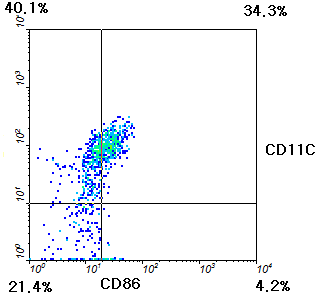 | 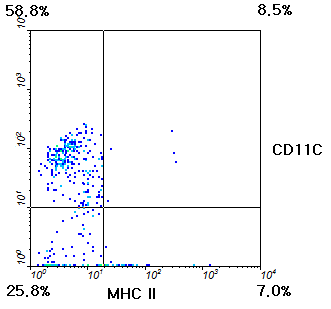 |
| 10  µg/ml | 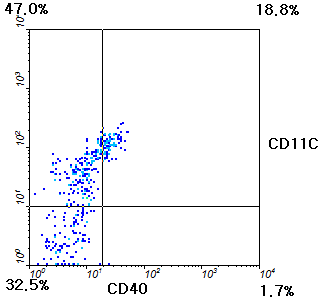 | 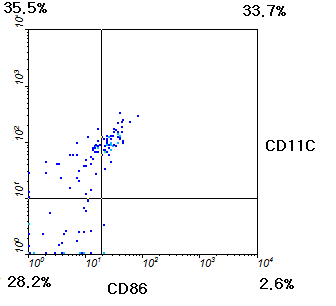 | 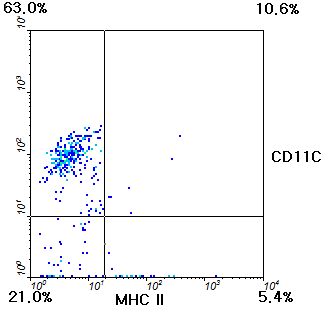 |
| 100  µg/ml | 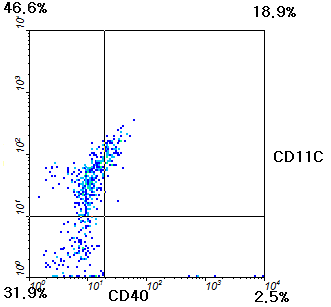 | 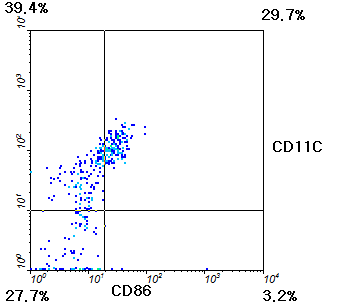 | 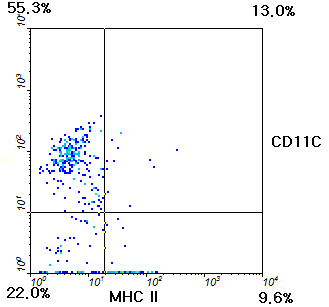 |
| DMSO | 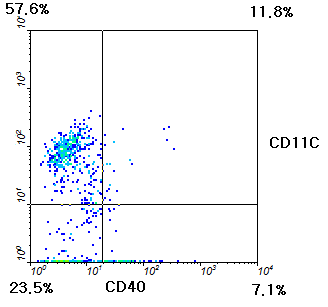 | 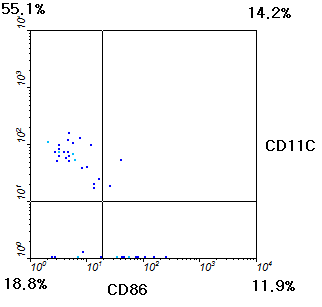 | 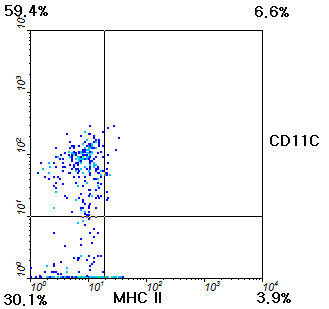 |
